# Mesalamine-induced photosensitivity – A case report and literature review^☆^

**DOI:** 10.1016/j.abd.2023.07.001

**Published:** 2023-12-08

**Authors:** Svetlana Popadic, Igor Kapetanovic, Aleksandra Sokic-Milutinovic

**Affiliations:** a Department of Dermatology and Venereology, Faculty of Medicine, University of Belgrade, Belgrade, Serbia; b Clinic of Dermatovenereology, University Clinical Center of Serbia, Belgrade, Serbia; c Department of Gastroenterology and Hepatology, Faculty of Medicine, University of Belgrade, Belgrade, Serbia; d Clinic for Gastroenterology and Hepatology, Clinical Center of Serbia, Belgrade, Serbia

*Dear Editor,*

We present a case of mesalamine-induced photosensitivity in a patient with Ulcerative Colitis (UC). To the best of our knowledge, this is the fourth case of mesalamine-induced photosensitivity reported so far.^1^, ^2^, ^3^

In April 2018, a 26-year-old male presented with diffuse erythema and erythematous papules on sun-exposed areas (Figure 1, Figure 2). Skin lesions appeared during the first sunny spring days (average UV index was 5 ‒ moderate). He has no previous history of sun sensitivity. In personal history, five weeks before admission to our department, he started treatment for UC. Treatment included oral and rectal forms of mesalamine alongside oral prednisolone and esomeprazole.

**Figure 1:**
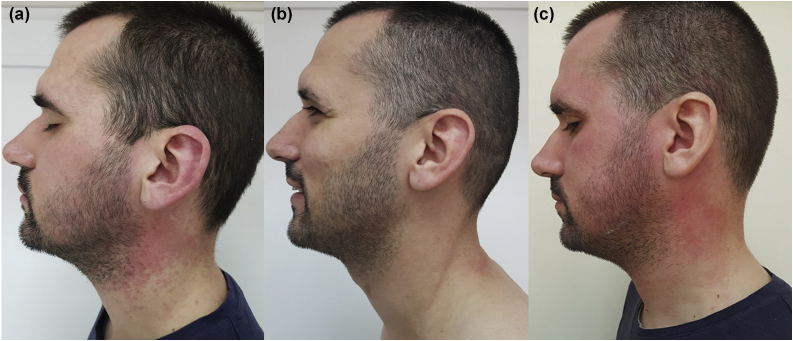
(A) Erythema and erythematous papules on the cheeks, auricles, and neck. (B) Face, auricles, and neck without skin lesions. (C) Erythema and erythematous papules on the scalp, face, auricles, and neck.

**Figure 2:**
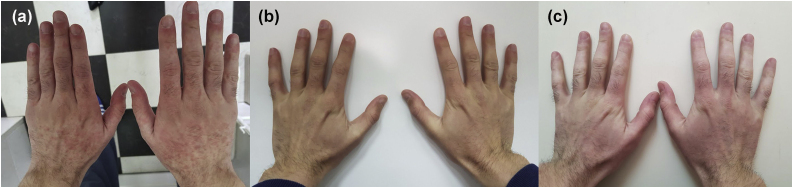
(A) Erythema and erythematous papules on dorsal side of the hands. (B) Dorsal side of the hands without skin lesions. (C) Erythema on dorsal side of the hands.

At admission routine laboratory analysis of blood and urine were within normal limits. Immunological analysis (IgG, IgM anti-cardiolipin antibodies, IgG anti-mitochondrial antibodies, IgG anti-nucleus antibodies, IgG anti-smooth muscle antibodies, IgG anti-liver kidney microsome type 1 antibodies, IgG anti-parietal antibodies, anti-Extractable Nuclear Antigen (ENA) antibody screen, IgG anti-dsDNA, C3 and C4) were all negative. HIV antigen/antibody, HBsAg, and anti-HCV were all negative. VDRL was negative. Lupus bend tests from sun-exposed and sun-protected areas were negative.

We suggested sun protection along with sun avoidance and initiated topical corticosteroid preparations with concomitant UC treatment (mesalamine 3 g/day, prednisolone 20 mg/day and esomeprazole 20 mg BID) for 4 days. The suggested treatment was without result, erythema was persistent. Due to suspicion that sun sensitivity was induced by mesalamine, mesalamine and topical corticosteroid treatment were discontinued and 20 mg of prednisolone alongside esomeprazole and sun protection remain the only treatment. During the following 4 days, he spent up to 1 hour/day in the sun and despite that, there was a complete regression of skin lesions (Figure 1, Figure 2).

We were interested on whether photosensitivity appears again in the absence of mesalamine so, we suggested unlimited sun exposure without sun protection, for the next 6 days (10 days in total without mesalamine). During those days were no signs of photosensitivity, but UC worsened and mesalamine re-challenge was approached. He took the first, reintroduced, dose of mesalamine and 12‒15 h later, he spent 30 minutes outside on a sunny morning. During those 30 minutes, erythema reappeared on sun-exposed areas. We established the diagnosis of mesalamine-induced photosensitivity (Figure 1, Figure 2). The gastroenterologist substituted mesalamine with azathioprine and prednisolone was tapered and stopped. UC has been in remission since October 2018, and as of April 2023, he has no manifestations of sun sensitivity.

Mesalamine is the preferred, safe, first-line treatment with proven efficacy in mild to moderate forms of UC^4^ with mild systemic absorption despite its delayed release mechanism.^5^ According to the literature mesalamine may be associated with skin rashes and pruritus.^4^, ^5^

Many drugs have been implicated in photosensitive reactions but mesalamine is not considered to be one.^6^, ^7^ Only one case of probable esomeprazole-induced photoallergic dermatitis has been reported so far.^8^ Cutaneous adverse drug reactions in sun-exposed areas may be phototoxic or photo-allergic type.^6^ Photo-toxicity appears rapidly after sun exposure because of light activation of the photosensitizing agent, while photoallergy skin lesion appears after 2‒3 days due to activation of cell-mediated immune response.^7^ Esomeprazole was not in the treatment protocol in other reported cases with mesalamine-induced photosensitivity.^1^, ^2^, ^3^ In our patient photosensitivity was not associated with esomeprazole intake. The proof for that is that he had no skin lesions while being treated with esomeprazole in periods when mesalamine was temporarily or permanently withdrawn. The prompt reappearance of erythema in our patient after mesalamine re-challenge indicates a phototoxic type of photosensitivity.

Among all published cases (Table 1.) our case is the only case with discontinuation of mesalamine treatment and a re-challenge test. In accordance with the Naranjo ADR probability scale,^9^ the strength of association between mesalamine and the development of (sun) photosensitivity in our patient was “definite” (total score 9).

**Table 1:** Cases of mesalamine induced photosensitivity reported in the literature.

	Patient age at presentation	Onset of symptoms after mesalamine introduction	Season at the time of onset of skin lesions	Symptom resolution after mesalamine discontinuation	Mesalamine re-exposure
**Our patient**	26	5 weeks	Spring	4 days	Yes
**Al-Niaimi et al.**	56	2‒3 months	Summer	10 days	No
**Cozzani et al.**	46	6 months	Summer	6 months	No
**Horiuchi et al.**	48	2 weeks	Spring	NA	NA

NA, Not applicable (mesalamine not discontinued).

In reported cases, the period of photosensitivity onset after mesalamine introduction ranged from 2 weeks to 6 months (Table 1). In our patient, erythema completely disappeared within 4 days after mesalamine was withdrawn similar to Al-Niaimi et al. reported resolution within 10 days.^1^ However, Cozzani et al. reported complete resolution 6 months after discontinuation of mesalamine.^2^ Interestingly Horiuchi et al. never withdrew mesalamine and reportedly symptoms were controlled by sun protection.

To conclude, we believe that dermatologists should be aware of photosensitivity as a possible mesalamine side effect, to avoid unnecessary clinical evaluations.

## Financial support

None declared.

## Author’s contributions

Svetlana Popadic: The study concept and design; data collection, or analysis and interpretation of data; statistical analysis; writing of the manuscript or critical review of important intellectual content; data collection, analysis, and interpretation; effective participation in the research guidance; intellectual participation in the propaedeutic and/or therapeutic conduct of the studied cases; critical review of the literature; final approval of the final version of the manuscript.

Igor Kapetanovic: Writing of the manuscript or critical review of important intellectual content; data collection, analysis and interpretation; final approval of the final version of the manuscript.

Aleksandra Sokic-Milutinovic: Intellectual participation in the propaedeutic and/or therapeutic conduct of the studied cases; critical review of the literature; final approval of the final version of the manuscript.

## Conflicts of interest

None declared.
